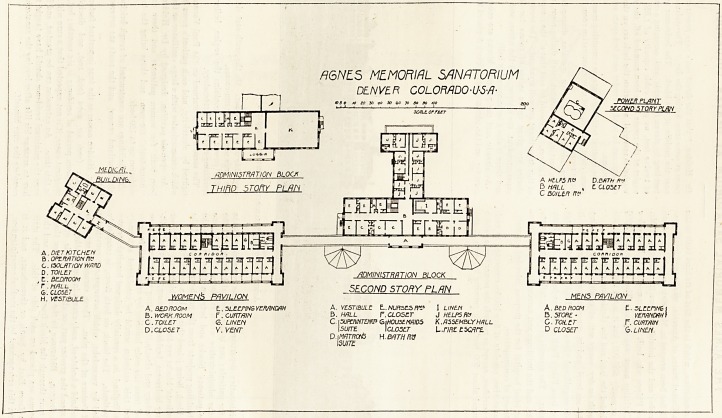# The Agnes Memorial Sanatorium at Denver, Colorado

**Published:** 1906-04-28

**Authors:** 


					74, THE HOSPITAL. Aran. 28, 1906.
A6NES MEMO/ML SANATORIUM PCrWCR^Ga^ORADO
DENVER COLORRDO-U-5-fJ?
to 20 iO So 60 fo So
5C/ILL OF riLCT
A. PUMP ROOM G. ENTRANCE
B.EN6INE - H.COAL
C.BOILER - I WISHES
D.LAUNDRY J.CREMATORY
L.STERILIZIN6 R1S K. SMOKE STACK
T. TOILET
raT-j*-
-vy^, L L L L L I I   I Lt|0i_|^c-|J ^ ^
A. COH3ULT/1TION ?*| conn/not? ? j"W
B. RECEPTION | I n 3 33 3 ^3 3 3^0'
r-. ruiT <z~r p \/n\si Hi I I I I ' I ' I l 1 ' 1 *1 ' I * I ' I f ffl
C\ CHEST EXAM
D . NOSE V THROAT
LAlu?l-?L? l?U u% l
sisawsr r j ***
r Lyp/ I tmamm LJ rl ' '"Ullill *?- ? Mf II I
6. dru& room FIRST STORY PLAN-
h! toilet womens pavilion- uu~~ mens pavilion
'i ' ^OTOIV/ELOPING A. BEDROOM E. 3LEEPINGVERANDAH A, VESTIBULE G SUPERip- L HELPONmfK A. RED ROOM F CURTAINS
Kv'Atipjii f B. WORK ROOM r. CURTAIN B. HALL TEND"' M. REFRIGERATOR B .WORK- G LINEN
C. TOILET S. LINEN C. OFFICE H. STAFF DINING R$ N./'/ttStfaf C. TOILET H '.BARBERSHOP
D. CLOSET V.VENT D. RECEPTION I. SERVING RV 0. VERANDA D. CLOSET V VENT
L. BOARD /fir d.mwe " P ENTRANCE C.. SLEEPING VERAHDAH
F. UBRRRY K. KITCHEN Q PASTRY
FOIl SECOND STOUT PLAN, SEE PAGE 76.
April 28, 190G. THE HOSPITAL.
THE ACNES MEMORIAL SANATORIUM AJ DENVER, COLORADO,.
This sanatorium, which is the first of its kind built in the
Western United States of America, was opened in the year
1904. It has been erected in Montelair, which is a suburb
of Colorado; and the inhabitants owe its existence to Mr.
Lawrence Phipps who built it as a memorial to his mother
whose Christian name was Agnes. The cost was 300,000
dollars, or nearly ?50,000; but it is not stated whether this
sum included the land and the furnishing or not.
It is built on dry, sandy soil, at an altitude of 5,400 feet
above the sea-level, and in consequence of this height it
commands magnificent views of the Rocky Mountain Range
extending from Pike's Peak on the one hand to Long's Peak
on the other. The grounds are beautifully laid out in lawns
and walks, and the buildings and surroundings should form
an almost ideal hospital for the purpose intended?namely,
4he treatment of consumptives.
The sanatorium consists of five blocks connected to each
other by corridors, except as regards the power house, which
3aas a tunnel about 500 feet long running between it and
the administrative department. Of,the remaining blocks
two are for patients; one is the medical building and the
other is the administrative which is placed almost in line
with the patients' blocks and between them, while the
snedical building projects from the north-west corner of the
women's block, being joined thereto by a corridor.
The patients' blocks are two stories high, and each floor
has twenty bedrooms, besides workroom, linen-room, bath-
room, lavatory and closets. Regarding the latter they are
not cut off from the main building by cross-ventilated pas-
sages, and this omission would be considered extremely
faulty in England, in a specially designed hospital for any
?class of patients. The block, on both floors, is completelv
surrounded by verandahs, which arrangement constitutes
the chief feature of the plan. These verandahs are wide
?enough to hold a bedstead, and, except in bad weather, the
patients spend the greater part, or almost the whole, of the
24 hours thereon. Reference to the plan will show that a
?corridor runs through the block from end to end, and that
the bedrooms are placed on each side of this. The main
elevation faces East by South or thereabouts, and it is
claimed for this arrangement by the medical superintendent,
V
Dr. Holden, " that the direct sun's rays penetrate into each
room at some time during the day." The staircase is placed
near the middle of the block. The bedrooms are fourteen
feet long by eleven feet wide, and, assuming a ceiling height
of twelve feet, we get a cubic capacity of 1,850 feet. The
angles of the rooms have been rounded off, the walls are of
washable material, and when the beds are on the verandahs
curtains can be used to render each bed-space separate from
the others.
The medical building contains consulting-room, reception-
room, chest, throat, and nose examination room, laboratory,
x-ray room, etc.
The administrative block contains dining-hall, board-
room, serving-room, kitchen, staff dining-room, and all the
usual offices. It should be said that all the component parts
of this department are well arranged. The second and third
stories are given up to suites of rooms for the medical
superintendent, the matron, and the nurses and domestics;
and there is also a large assembly hall.
As already said the patients' blocks are connected by a
corridor, and the administrative block is placed to the north-
west side of the corridor. On the south-east side there are
two large semicircular sun-rooms.
The power house contains the engine, boiler, laundry,
sterilising-room, and crematory.
The sanatorium is warmed on the Paul Vacuum System,
lighted by electricity, and each patient's room has a'shaft
which communicates with a large ventilator on the roof.
An open-air pavilion, designed by Dr. Holden, has also
been built for women patients; and there are modern
" house-tents" for men; and these bring the total accom-
modation up to 150 beds.
The buildings are constructed of rough brickwork faced
with Portland cement, and the roofs are covered with red
tiles.
Dr. Holden names as the four important factors of treat-
ment : fresh air, good food, rest, and discipline. The
minimum period of residence is six months; but, except in
incipient cases, it is not expected that a cure will be effected
in this time. An extension can be granted, and in any case
even the minimum residence should have taught the patient
how to arrange his life after leaving the sanatorium, and so
complete the cure himself.
SBSBSpSK SMS &S5B BSB ?
v v;**5* rf?e - * _;: v V :;;- - ? ?, -;
" V'i? ? >. , -
rW# -??*.?=.? ?"-^???'^ ?-
Agnes Memorial Sanatorium, Denver, Colorado.
76 THE HOSPITAL. April 28, 1906.
A6NE.5 MEMORIAL SANATORIUM
DENVER COLOMDO-U-S-/J-
10 3 0 fO to SO iO 50 bo TO do SO /CO i
6. CLOSET
H. VESTIBULE
/JDMINISTRATION BLOCK
A HELPS flc D.BfiTH ft*
B HALL , t CLOSET
C boiler
A.DIET KITCHEN fl ^ con^oo^ ' I L--
B . OPERATION ftP | [333313333313321 V/
C. ISOLATION WARD | I ,AJ A| A| /I A" | A( AH /Tl *] aH *1 /TJ I
?BEDROOM Ty jeJ !^i i ; i j. _1 1 ' ;
' SECOND STORY PLAN
WOMEN?, PAVILION MENS PAVILION
A BEDROOM E.. SLEEPING VERANDAH A. VESTIBULE L. NURSES fit* I LINEN A. BEDROOM t. SLEEPING I
B. WORK ROOM r. CURTAIN V.HALL P. CLOSET J HELPS Ha B .STORE- VERANDAH)
C. TOILET G. LINEN C. \SUPWNTEtW GiHOUSEMHIDS K. ASSEMBLY H/JLL C. TOILtT F. CURTAIN
D.CLOSET V. VENT I SUITE I CLOSET L.f/fif E SCARE D CLOSET 6. LINEN.
D.t MATRCM5 H.BATH W
ISU/TE

				

## Figures and Tables

**Figure f1:**
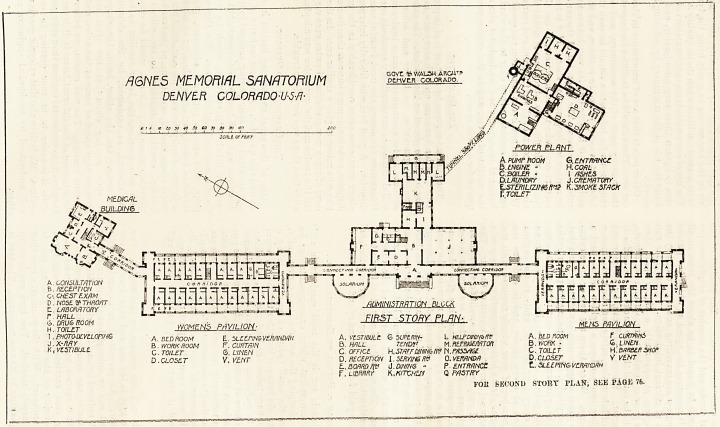


**Figure f2:**
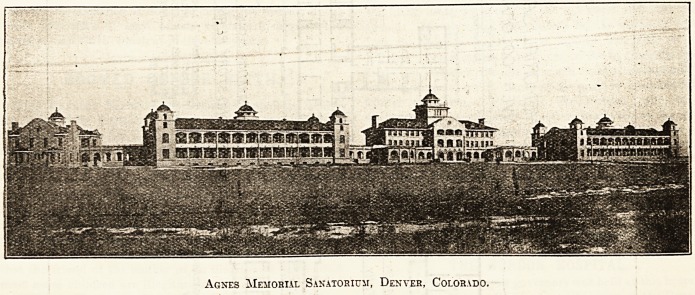


**Figure f3:**